# Cerebrospinal fluid phosphorylated tau, visinin-like protein-1, and chitinase-3-like protein 1 in mild cognitive impairment and Alzheimer’s disease

**DOI:** 10.1186/s40035-018-0127-7

**Published:** 2018-09-10

**Authors:** Hua Zhang, Kok Pin Ng, Joseph Therriault, Min Su Kang, Tharick A. Pascoal, Pedro Rosa-Neto, Serge Gauthier, the Alzheimer’s Disease Neuroimaging Initiative

**Affiliations:** 1 Department of Neurology, the First Affiliated Hospital of Chongqing Medical University, Chongqing, 400016 China; 2 Department of Neurology, National Neuroscience Institute, Singapore, Singapore; 3 The McGill University Research Centre for Studies in Aging, McGill University, Montreal, Canada

**Keywords:** Alzheimer’s disease, Amyloid-β, Chitinase-3-like protein 1, Phosphorylated tau, Visinin-like protein-1

## Abstract

**Background:**

Visinin-like protein-1 (VILIP-1) and chitinase-3-like protein 1 (CHI3L1 or YKL-40) in cerebrospinal fluid (CSF) are newly discovered markers indicating neuronal damage and microglial activation, respectively. Phosphorylated tau (p-tau) reflects the neuropathology of Alzheimer’s disease (AD) and is useful as diagnostic markers for AD. However, it is unknown whether these biomarkers have similar or complementary information in AD.

**Methods:**

We stratified 121 participants from the Alzheimer’s Disease Neuroimaging Initiative (ADNI) database into cognitively normal (CN), stable mild cognitive impairment (sMCI), progressive MCI (pMCI), and dementia due to AD. Analysis of covariance (ANOVA) and chi-square analyses, Spearman correlation, and logistic regression models were performed to test the demographic, associations between biomarkers, and diagnostic accuracies, respectively. Linear mixed-effects models were used to evaluate the effects of CSF amyloid-β (Aβ) on above biomarkers within diagnostic groups, the combination of diagnostic group and Aβ status as predictor, and CSF biomarkers as predictors of AD features, including cognition measured by Mini–Mental State Examination (MMSE) and brain structure and white matter hyperintensity (WMH) measured by magnetic resonance imaging (MRI).

**Results:**

P-tau, VILIP-1, and YKL-40 were all predictors of AD diagnosis, but combinations of biomarkers did not improve the diagnostic accuracy (AUC 0.924 for p-tau, VILIP-1, and YKL-40) compared to p-tau (AUC 0.922). P-tau and VILIP-1 were highly correlated (*r* = 0.639, *p* < 0.001) and strongly associated with Aβ pathology across clinical stages of AD, while YKL-40 was correlated with Aβ pathology in CN and AD groups. VILIP-1 was associated with acceleration of cognitive decline, hippocampal atrophy, and expansion of ventricles in longitudinal analyses. YKL-40 was associated with hippocampal atrophy at baseline and follow-up, while p-tau was only associated with worsening WMH at baseline.

**Conclusions:**

CSF levels of p-tau, VILIP-1, and YKL-40 may have utility for discriminating between cognitively normal subjects and patients with AD. Increased levels of both VILIP-1 and YKL-40 may be associated with disease degeneration. These CSF biomarkers should be considered for future assessment in the characterization of the natural history of AD.

**Electronic supplementary material:**

The online version of this article (10.1186/s40035-018-0127-7) contains supplementary material, which is available to authorized users.

## Background

Alzheimer’s disease (AD) is a progressively neurodegenerative disorder that is characterized by extracellular depositions of amyloid-β (Aβ), intracellular neurofibrillary tangles of hyperphosphorylated tau, and neuronal loss [1, 2]. The pathophysiological abnormalities that characterize AD precede clinical symptoms by many years and can be quantified in vivo using biomarkers. Based on the nature of the pathophysiology that each measures, the “A/T/N” system for biomarkers has been proposed, and Aβ is first, followed by tau, then neurodegeneration [3]. Over the past decade, inflammatory factors and other proteins have been proposed to further characterize the AD pathophysiological process [4]. It is important to study the ability of these biomarkers to make accurate diagnoses, which can permit for population enrichment of clinical trials [5].

Visinin-like protein-1 (VILIP-1), a calcium-mediated neuronal injury biomarker, has been shown to have diagnostic and prognostic value in distinguishing individuals with symptomatic AD from controls [6–10]. Therefore, these studies suggest that VILIP-1 may be useful for AD pathophysiology, but evidence is limited. Finding an association between VILIP-1 and AD pathophysiology may offer another important neurodegeneration biomarker specific for early diagnosis of AD.

A growing body of research suggests that the immune system is involved early in the pathogenesis of AD [11–13], with cytokines, chemokines, and other inflammatory mediators increased in the cerebrospinal fluid (CSF) or plasma of AD patients. Chitinase-3-like protein 1 (CHI3L1 or YKL-40), a 39 kDa glycoprotein homologue to chitinase, is one of the proteins that has been frequently measured in body fluids as a surrogate marker of neuroinflammation in AD [14, 15]. Furthermore, many studies [16–21], but not all [22], have found increased levels of CSF YKL-40 in AD patients.

However, it is not clear whether p-tau, VILIP-1, and YKL-40 provide independent or complementary information about AD and whether combinations of p-tau, VILIP-1, and YKL-40 increase the diagnostic accuracy for AD and mild cognitive impairment (MCI). Here, in a prospective longitudinal study, we aim to test the hypotheses that (1) combinations of p-tau, VILIP-1, and YKL-40 increase the diagnostic accuracy for AD and MCI; (2) p-tau, VILIP-1, and YKL-40 have different correlations with Aβ pathology and with different clinical stages of AD; and (3) p-tau, VILIP-1, and YKL-40 have different correlations with other AD features, including cognitive decline, cerebral atrophy, and white matter hyperintensities (WMH).

## Methods

### Database description and study participants

Data used in the preparation of this article were obtained from the Alzheimer’s Disease Neuroimaging Initiative (ADNI) database (adni.loni.usc.edu). The ADNI was launched in 2003 as a public-private partnership led by principal investigator Michael W. Weiner, MD. The primary goal of ADNI has been to test whether serial magnetic resonance imaging (MRI), positron emission tomography (PET), other biological markers, and clinical and neuropsychological assessment can be combined to measure the progression of MCI and early AD.

For the present study, we selected 121 ADNI-GO/2 participants who met the criteria for AD (*n* = 18), amnestic MCI (*n* = 71), and cognitively normal (CN, *n* = 32). Participants with MCI were divided into stable MCI (sMCI, *n* = 24) and progressive MCI (pMCI, *n* = 47) based on whether they converted to AD during follow-up. We defined probable AD as those who fulfill the National Institute of Neurological and Communicative Disorders and Stroke and the Alzheimer’s Disease and Related Disorders Association (NINCDS/ADRDA) criteria, Mini–Mental State Examination (MMSE) scores between 20 and 26 and a clinical dementia rating of 1.0 [23]. We defined MCI as those who had a MMSE score of 20–26, a clinical dementia rating of 0.5, subjective and objective memory loss, and absence of other neuropsychiatric disorders. We further stratified the MCI participants into sMCI if they did not progress to AD during at least 2 year of follow-up, and pMCI if they progress to AD at any time during the follow-up. We defined CN as those with a MMSE score of 27 or higher, a clinical dementia rating 0 and absence of any neuropsychiatric diagnosis, MCI and dementia. We excluded the subjects who were diagnosed as CN at baseline, but converted to MCI or AD during follow-up; subjects who were diagnosed as MCI at baseline, but reverted to CN during follow-up; subjects who were diagnosed as AD at baseline, but reverted to MCI during follow-up. (Further information about the inclusion/exclusion criteria may be found at www.adni-info.org (accessed February 2018).

### Standard protocol approvals, registrations, and patient consents

The ADNI study was approved by the Institutional Review boards of all of the participating institutions. All participants provided informed consent at each site.

### Cognitive assessment

Cognitive assessment was performed by certified raters using standardized ADNI protocols. The MMSE was used to measure the global cognition of the study participants. Longitudinal cognitive data from up to 13 time points, from baseline to 120 months follow up, were analyzed. The data used in this study was obtained from the ADNI files “MMSE.csv”, (accessed February 2018).

### CSF analyses

CSF Aβ42, t-tau, and p-tau at threonine 181 were measured by using the multiplex xMAP Luminex platform (Luminex Corp, Austin, TX) and Innogenetics INNO-BIA AlzBio3 (Innogenetics, Ghent, Belgium) immunoassay reagents. Those procedures have been described previously [24]. A sandwich ELISA was developed using the Erenna® immunoassay system to measure CSF VILIP-1 [8]. CSF YKL-40 was measured using the MicroVueYKL-40 ELISA assay [17]. All of the CSF data used in this study were obtained from the ADNI files “UPENNBIOMK5–8.csv” and “FAGANLAB_07_15_2015.csv.” Further details of ADNI methods for CSF acquisition and measurements and quality control procedures can be found at www.adni-info.org, (accessed February 2018).

### Neuroimaging methods

The neuroimaging data, including the hippocampal and ventricular volume and the white matter hyperintensity (WMH) on MRI, were obtained from the ADNI files “FOXLABBSI_08_04_17.csv”, “UCSDVOL.csv”, and “UCD_ADNI1_WMH.csv” (accessed February 2018). All the imaging data were selected at 5 time points: baseline, 6, 12, 24, and 36 months. The neuroimaging methods used by ADNI have been reported previously [25]. To evaluate neurodegeneration, we used both hippocampal and ventricular volumes. In addition, we analyzed WMH volume, which is a surrogate marker for cerebrovascular disease load [26]. Further details for ADNI image acquisition and processing can be found at www.adni-info.org/methods (accessed February 2018).

### Statistical methods

Analysis of variance (ANOVA) and chi-square tests were used to test for significant differences between groups on demographics for continuous and categorical measurements respectively. Spearman correlation was performed to test associations between CSF p-tau, VILIP-1, YKL-40, and Aβ42.

Logistic regression models were used to test the diagnostic accuracies of each biomarker at baseline against clinical diagnostic criteria for CN versus AD and sMCI versus pMCI. Overall diagnostic accuracy (area under the receiver operating characteristic curve, AUC) was also obtained from Receiver operating characteristic curve (ROC) analyses for each biomarker. The bootstrap method was used to further examine the differences of the AUCs between each biomarker. Classification tables were extracted from the logistic regression models to quantify correct classifications of CN, AD, sMCI, and pMCI, using a 50% threshold of predicted probability. The above models were adjusted for age, gender, education, and CSF Aβ42.

Linear mixed-effects were used to test the association between p-tau, VILIP-1 and YKL-40 with Aβ status within each diagnostic group. Each diagnostic group was dichotomized using a previously established cutoff of CSF Aβ42 (< 192 pg/ml) [24], and compared Aβ-negative (Aβ-) versus Aβ-positive (Aβ+). The models were adjusted for age, sex, and education. All models included a random intercept. The estimates (β-coefficients (*p* values)) for the Aβ statuses were presented in the table. The columns marked “difference” test whether the comparison differed between two biomarkers.

The association between CSF biomarkers and different AD features (including MMSE, hippocampal volume, ventricular volume, and WMH) at baseline were tested using linear mixed-effects models. The predictors were biomarker, age and sex. Cognition was also adjusted for education and volume measures for intracranial volume. Data are estimates (β-coefficients) from linear mixed-effects models, with 95% confidence intervals. The estimates are the main effects of the biomarkers, capturing the effects at study baseline.

Finally, linear mixed-effects models were also used to evaluate CSF biomarkers as predictors of different AD features, including MMSE, hippocampal volume, ventricular volume, and WMH. The predictors were a biomarker by time (years) interaction, age, sex, diagnosis (CN, sMCI, pMCI, and AD), and all main effects. Cognitive measures were adjusted for years of education and hippocampal volume was adjusted for intracranial volume. The models included random intercepts and slopes and an unstructured covariance matrix for the random effects. Models were tested separately for Aβ- and Aβ + subject. Residuals followed a homoscedastic distribution and data met the model’s assumption of linearity. Statistical significance was defined as *p* < 0.05 for all analyses. All statistics were done using SPSS version 20 and R (v. 3.4.2).

## Results

### Demographic results

Table 1 lists the demographic characteristics of all the participants. There were no differences in age and education among the groups. There was no significant difference in sex between CN, sMCI, and pMCI groups, but the prevalence of female in AD group was significantly higher than that in other groups. APOE ε4 carriership was more common in pMCI and AD than in CN and sMCI. The follow-up time of participants in CN group was significantly longer than that in other groups, especially the AD group.

**Table 1:** Demographics of subjects

Characteristics	CN (*n* = 32)	sMCI (*n* = 24)	pMCI (*n* = 47)	AD (*n* = 18)
Age, years [mean (SE)]	76.0 (1.0)	76.7 (1.1)	73.1 (1.0)	74.3 (1.6)
Gender, male [n (%)]	19 (59.4%)	17 (70.8%)^d^	33 (70.2%)^d^	7 (38.9%)
Education, years [mean (SE)]	16.1 (0.6)	16.6(0.5)	15.9 (0.4)	15.2 (0.7)
APOE ε4+ [n (%)]	5 (15.6%)^c, d^	8 (33.3%)^c, d^	28 (59.6%)^a, b^	13 (72.2%)^a, b^
MMSE at baseline, [mean (SE)]	29.2 (0.2)^b, c, d^	27.2 (0.3)^a, d^	26.6 (0.2)^a, d^	24.2 (0.5)^a, b, c^
MMSE at 24 m, [mean (SE)]	29.3 (0.2) ^b, c, d^	26.7 (0.5)^a, c, d^	24.3 (0.5)^a, b, d^	18.1 (1.6)^a, b, c^
Follow-up, years, [mean (SE)]	7.0 (0.4)^b, c, d^	4.7 (0.5)^a, d^	5.7 (0.4)^a, d^	2.6 (0.2)^a, b, c^

*P* values indicate the values assessed with analyses of variance for each variable except gender and APOE ε4, where a contingency chi-square was performed. Post hoc analysis provided significant differences between groups: ^a^from CN; ^b^from sMCI; ^c^from pMCI; ^d^from AD

*Abbreviations*: *SE* Standard error, *APOE* apolipoprotein E, *MMSE* Mini-mental State Examination, *CN* cognitively normal, *sMCI* stable mild cognitive impairment, *pMCI* progressive mild cognitive impairment, *AD* Alzheimer’s disease

### Baseline levels of CSF biomarkers

CSF Aβ42 levels were, as expected, lower in sMCI, pMCI, and AD groups compared with those who were cognitively normal, and lower in pMCI and AD compared with sMCI, as shown in Table 2. CSF p-tau was higher in both pMCI and AD groups compared with CN and sMCI groups, but there were no differences between pMCI and AD groups. CSF t-tau was higher in pMCI and AD groups compared with CN subjects, and higher in AD compared with pMCI. Neither t-tau nor p-tau was significantly different between CN and sMCI groups. Similar to the levels of CSF p-tau, mean baseline VILIP-1 levels were higher in both pMCI and AD groups compared with CN and sMCI groups. However, there was no significant difference in levels of VILIP-1 between pMCI and AD groups and between CN and sMCI groups. Mean baseline levels of YKL-40 in AD participants were higher than those in CN, sMCI, and pMCI, but the differences were not statistically significant.

**Table 2:** Baseline levels of CSF biomarker

Characteristics	CN (*n* = 32)	sMCI (*n* = 24)	pMCI (*n* = 47)	AD (*n* = 18)
Aβ42, pg/ml [mean (SE)]	226.1 (9.3) ^b, c, d^	178.5 (10.9)^a, c, d^	147.5 (7.0)^a, b^	136.1 (6.4)^a, b^
T-tau, pg/ml [mean (SE)]	64.3 (3.7)^c, d^	87.2 (10.8)^d^	107.8 (7.2)^a, d^	153.2 (19.1)^a, b, c^
P-tau-181, pg/ml [mean (SE)]	22.2 (1.6)^c, d^	28.9 (3.1)^c, d^	39.5 (2.4)^a, b^	45.8 (3.9)^a, b^
VILIP-1, pg/ml [mean (SE)]	133.0 (6.7)^c, d^	146.0 (10.6)^c, d^	184.3 (9.4)^a, b^	189.7 (16.6)^a, b^
YKL-40, ng/ml [mean (SE)]	397.2 (25.7)	384.0 (28.1)	400.7 (19.4)	471.9 (39.5)

*P* values indicate the values assessed with analyses of variance for each variable. Post hoc analysis provided significant differences between groups: ^a^from CN; ^b^from sMCI; ^c^from pMCI; ^d^from AD

*Abbreviations*: *SE* Standard error, *VILIP-1* Visinin-like protein-1, *YKL-40* Chitinase-3-like protein 1, *CN* cognitively normal, *sMCI* stable mild cognitive impairment, *pMCI* progressive mild cognitive impairment, *AD* Alzheimer’s disease

### Correlations between CSF p-tau, VILIP-1, YKL-40, and Aβ42

**Fig. 1:**
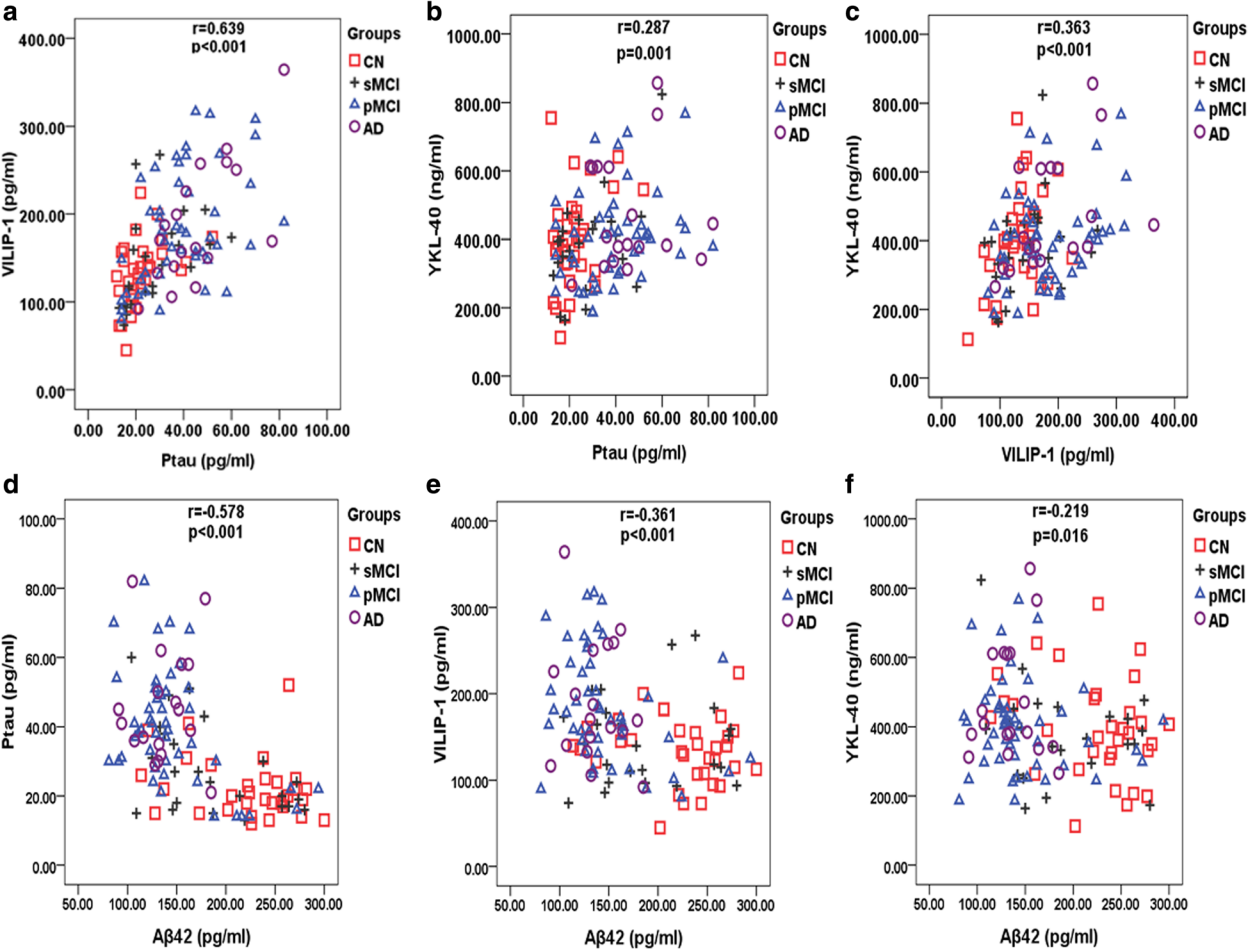
Correlations between CSF P-tau, VILIP-1, YKL-40, and Aβ42. **a**-**c** Correlations between CSF P-tau, VILIP-1, and YKL-40. **d**–**f** Correlations between these biomarkers and CSF Aβ42. Associations are shown for Spearman correlations. Abbreviations: VILIP-1, Visinin-like protein-1; YKL-40, Chitinase-3-like protein 1; CN, cognitively normal; sMCI, stable mild cognitive impairment; pMCI, progressive mild cognitive impairment; AD, Alzheimer’s disease

CSF p-tau, VILIP-1, and YKL-40 were correlated with each other (Fig. 1a–c), and the correlation between p-tau and VILIP-1 was especially strong (*r* = 0.639, *p* < 0.001). As shown in Fig. 1d–f, p-tau, VILIP-1, and YKL-40 correlated with Aβ42 negatively, and the strongest correlation was between p-tau and Aβ42 (*r* = − 0.578, *p* < 0.001).

### The effects of CSF p-tau, VILIP-1, and YKL-40 on diagnostic accuracy

**Fig. 2:**
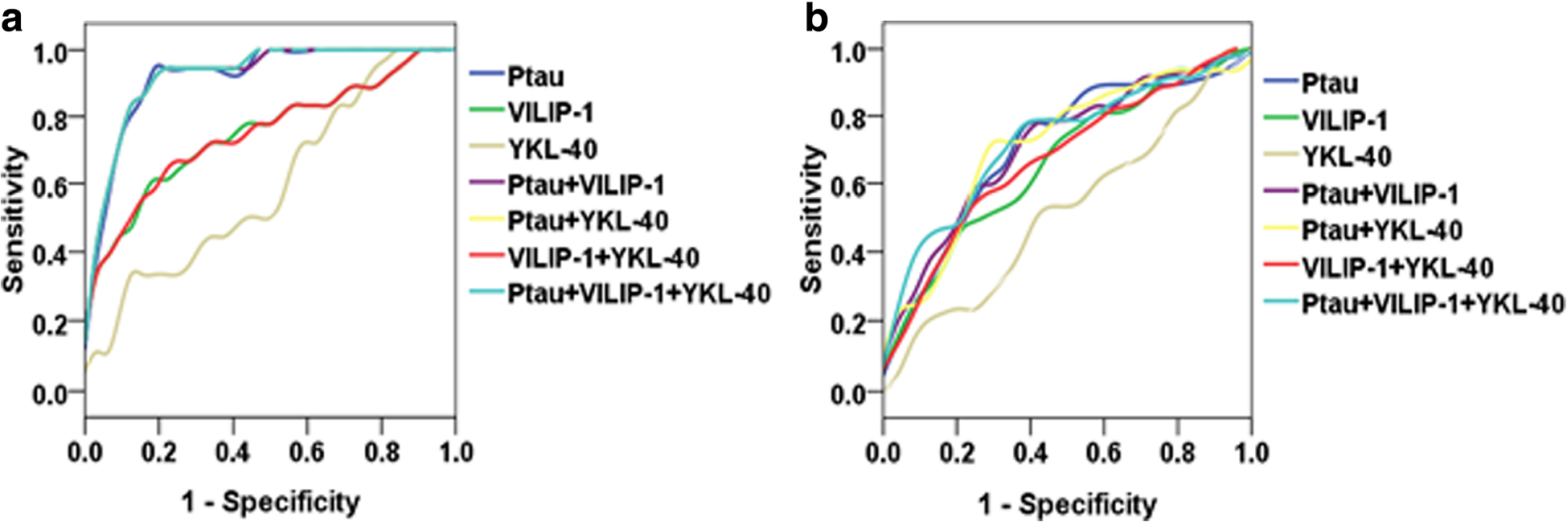
ROC analyses. **a** ROC for the diagnostic utility of CSF biomarkers and ratios in CN versus AD. **b** ROC for the diagnostic utility of CSF biomarkers and ratios in sMCI versus pMCI. Abbreviations: VILIP-1, Visinin-like protein-1; YKL-40, Chitinase-3-like protein 1; CN, cognitively normal; sMCI, stable mild cognitive impairment; pMCI, progressive mild cognitive impairment; AD, Alzheimer’s disease

Logistic regression models were used to test the diagnostic accuracy for CN versus AD and for sMCI versus pMCI. P-tau and VILIP-1, but not YKL-40, were significant predictors of AD. However, p-tau had the higher accuracy for single predictors (AUC 0.922). After adjusted age, gender, and education, YKL-40 was significant predictor for AD (β = 1.005, *p* = 0.048) (Additional file 1: Table S1). The highest accuracy was achieved using all biomarkers together (AUC 0.924), but the differences were not statistically significant between using biomarkers together and using p-tau alone (The upper part of Table 3 and Fig. 2a).

**Table 3:** Diagnostic accuracy of CSF P-tau, VILIP-1, and YKL-4

Groups	Model	P-tau	VILIP-1	YKL-40	AUC (95% CI)
CN vs AD	P-tau only	**1.177 (*p*** **< 0.001)**			**0.**922 (0.847–0.997)^b, c, f^
	VILIP only		**1.023 (*p*** **= 0.005)**		0.753 (0.604–0.903)^a, c, d, e, g^
	YKL-40 only			1.003 (*p* = 0.112)	0.601 (0.437–0.764)^a, b, d, e, f, g^
	P-tau & VILIP	**1.175 (*p*** **= 0.001)**	1.001 (*p* = 0.943)		0.922 (0.847–0.997) ^b, c, f^
	P-tau & YKL-40	**1.178 (*p*** **< 0.001)**	1.000 (*p* = 0.921)		0.924 (0.850–0.997) ^b, c, f^
	VILIP & YKL-40		**1.022 (*p*** **= 0.009)**	1.000 (*p* = 0.886)	0.753 (0.604–0.903) ^a, c, d, e, g^
	P-tau & VILIP & YKL-40	**1.176 (p7 = 0.001)**	1.001 (*p* = 0.912)	1.000 (*p* = 0.896)	0.924 (0.850–0.997) ^b, c, f^
sMCI vs pMCI	P-tau only	**1.056 (*p*** **= 0.007)**			0.708 (0.580–0.836)^c^
	VILIP only		**1.011 (*p*** **= 0.019)**		0.666 (0.5.5–0.797)^c^
	YKL-40 only			1.001 (*p* = 0.617)	0.524 (0.381–0.667) ^a, b, d, e, f, g^
	P-tau & VILIP	1.043 (*p* = 0.064)	1.006 (*p* = 0.279)		0.715 (0.591–0.840)^c^
	P-tau & YKL-40	**1.061 (*p*** **= 0.006)**		0.999 (*p* = 0.544)	0.716 (0.590–0.843)^c^
	VILIP & YKL-40		**1.012 (*p*** **= 0.020)**	0.999(*p* = 0.741)	0.676 (0.547–0.806)^c^
	P-tau & VILIP & YKL-40	**1.047 (*p*** **= 0.049)**	1.007 (*p* = 0.231)	0.998 (*p* = 0.419)	0.727 (0.607–0.847)^c^

For AUC, the letters a-g indicate significant differences versus other models: P-tau (a), VILIP-1 (b), YKL-40 (c), P-tau & VILIP-1 (d), P-tau & YKL-40 (e), VILIP-1 & YKL-40 (f), P-tau & VILIP-1 & YKL-40 (g). Bold values indicate significant associations

*Abbreviations*: *VILIP-1* Visinin-like protein-1, *YKL-40* Chitinase-3-like protein 1, *CN* cognitively normal, *sMCI* stable mild cognitive impairment, *pMCI* progressive mild cognitive impairment, *AD* Alzheimer’s disease

Similarly, p-tau and VILIP-1, but not YKL-40, were significantly individual predictors for sMCI versus pMCI. After adjusting for age, gender, and education, YKL-40 was also not a significant predictor for sMCI versus pMCI (Additional file 1: Table S1). Biomarkers combination did not have significant higher AUC than the model using only p-tau (The lower part of Table 3 and Fig. 2b).

After adjusting for age, gender, and education, although these models had higher AUCs than the basic models without covariates, the overall trends were similar (Additional file 1: Table S1).

### The effects of CSF p-tau, VILIP-1, and YKL-40 on classification of different groups

Based on a threshold of 50% for the predicted probability of the logistic regression models, classification tables were extracted from the models (Table 4). For single factor, p-tau had the highest correct classification for AD (14 of 18), followed by VILIP-1 (8 of 18) and YKL-40 (2 of 18). P-tau, VILIP-1, and YKL-40 had the same classification ability for CN. Comparing with p-tau, combination of biomarkers did not increase the classifications of CN and AD. However, the correct classification of AD was significantly increased after adjusting for age, gender, education, and Aβ42, and did not reduce the classifications of CN (The left part of Table 4).

**Table 4:** The effects of CSF P-tau, VILIP-1, and YKL-40 on classification

Model	CN vs AD		sMCI vs pMCI	
	Correct	Correct	Correct	Correct
	CN (%)	AD (%)	sMCI (%)	pMCI (%)
No covariates				
P-tau only	29/32 (90.6%)	14/18 (77.8%)	11/24 (45.8%)	42/47 (89.4%)
VILIP only	29/32 (90.6%)	8/18 (44.4%)	6/24 (25.0%)	43/47 (91.5%)
YKL-40 only	29/32 (90.6%)	2/18 (11.1%)	0/24 (0%)	47/47 (100%)
P-tau & VILIP	29/32 (90.6%)	13/18 (72.2%)	9/24 (37.5%)	39/47 (83.0%)
P-tau & YKL-40	29/32 (90.6%)	14/18 (77.8%)	10/24 (41.7%)	40/47 (85.1%)
VILIP & YKL-40	30/32 (93.8%)	8/18 (44.4%)	5/24 (20.8%)	42/47 (89.4%)
P-tau & VILIP & YKL-40	29/32 (90.6%)	14/18 (77.8%)	10/24 (41.7%)	39/47 (83.0%)
Adjusted for age, gender, education, and Aβ42				
P-tau only	29/32 (90.6%)	16/18 (88.9%)	10/24 (41.7%)	41/47 (87.2%)
VILIP only	27/32 (84.4%)	15/18 (83.3%)	9/24 (37.5%)	41/47 (87.2%)
YKL-40 only	28/32 (87.5%)	13/18 (72.2%)	10/24 (41.7%)	42/47 (89.4%)
P-tau & VILIP	30/32 (93.8%)	17/18 (94.4%)	12/24 (50%)	42/47 (89.4%)
P-tau & YKL-40	30/32 (93.8%)	16/18 (88.9%)	10/24 (41.7%)	42/47 (89.4%)
VILIP & YKL-40	28/32 (87.5%)	15/18 (83.3%)	9/24 (37.5%)	41/47 (87.2%)
P-tau & VILIP & YKL-40	30/32 (93.8%)	17/18 (94.4%)	11/24 (45.8%)	40/47 (85.1%)

Classification tables from logistic regression models using a threshold of 50% for predicted probabilities

*Abbreviations*: *VILIP-1* Visinin-like protein-1, *YKL-40* Chitinase-3-like protein 1, *CN* cognitively normal, *sMCI* stable mild cognitive impairment, *pMCI* progressive mild cognitive impairment, *AD* Alzheimer’s disease

For sMCI versus pMCI, CSF biomarkers alone or in combination had high classification of pMCI and poor classification of sMCI. Adjusting for age, gender, education, and Aβ42 improved the classification of sMCI in some models, such as VILIP-1 only, YKL-40 only, P-tau & VILIP-1, and VILIP-1 & YKL-40 (Table 4, right side).

### Associations between biomarkers, clinical diagnosis, and Aβ pathology

**Fig. 3:**
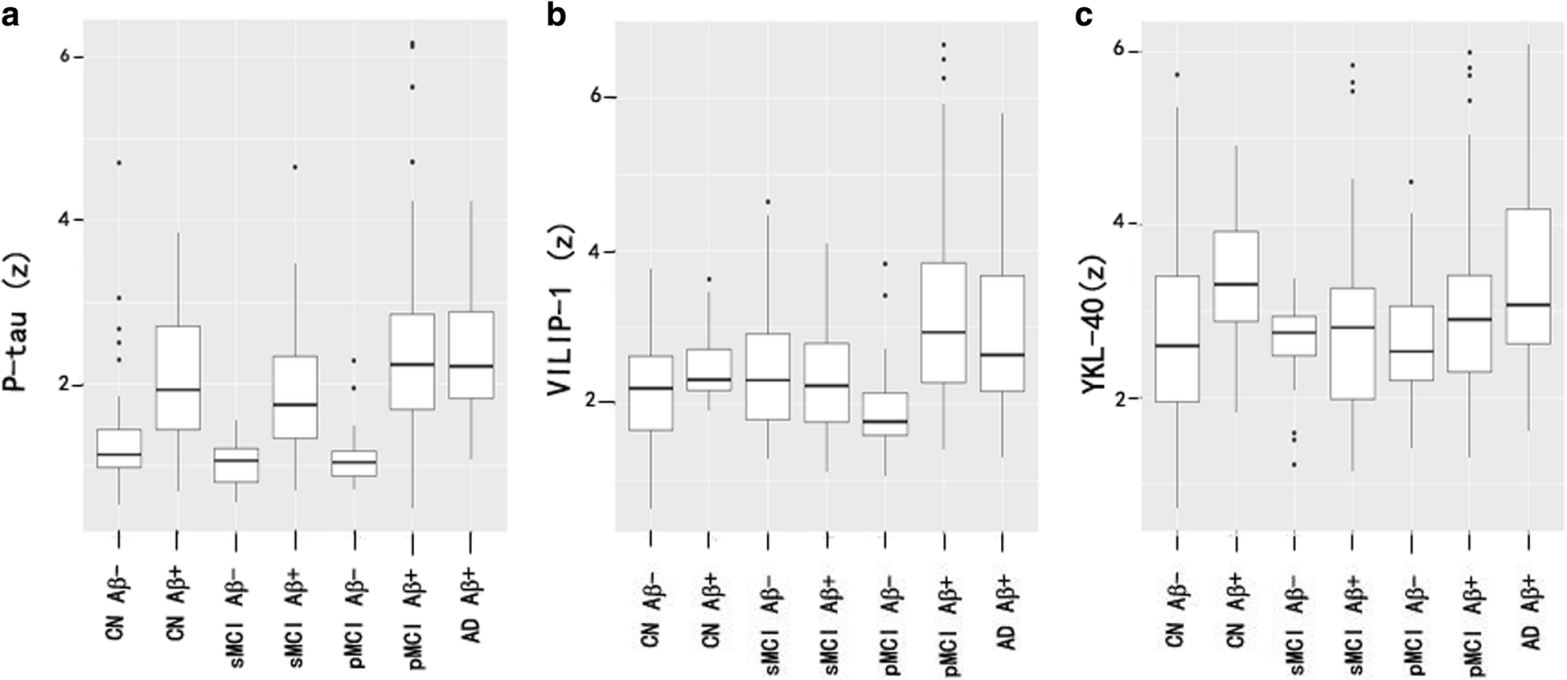
Biomarkers by clinical diagnosis and Aβ pathology. **a**–**c** CSF P-tau, VILIP-1, and YKL-40 in different combinations of clinical diagnosis and Aβ pathology. Biomarker levels are standardized to z-scores. Abbreviations: VILIP-1, Visinin-like protein-1; YKL-40, Chitinase-3-like protein 1; CN, cognitively normal; sMCI, stable mild cognitive impairment; pMCI, progressive mild cognitive impairment; AD, Alzheimer’s disease

Based on the combination of diagnosis and Aβ pathology, subjects were grouped as CN Aβ- (*n* = 24), CN Aβ + (*n* = 8), sMCI Aβ- (*n* = 8), sMCI Aβ + (*n* = 16), pMCI Aβ- (*n* = 6), pMCI Aβ + (*n* = 41), and AD Aβ + (*n* = 18). There were no AD participants who were Aβ- in this study. P-tau, VILIP-1, and YKL-40 were compared between these groups (Table 5 and Fig. 3). Between Aβ- and Aβ + subjects within diagnosis, Aβ + was correlated with increased p-tau in all groups (Table 5 and Fig. 3a) and with increased VILIP-1 in pMCI group (Table 5 and Fig. 3b) and with increased YKL-40 in CN group (Table 5 and Fig. 3c).

**Table 5:** CSF P-tau, VILIP-1, and YKL-40 across clinical diagnoses and Aβ pathology

Different groups	P-tau	VILIP-1	YKL-40	Difference P-tau vs VILIP-1	Difference P-tau vs YKL-40	Difference VILIP-1 vs YKL-40
Associations between neurodegeneration biomarkers and Aβ pathology within diagnostic group						
CN Aβ^−^ vs CN Aβ^+^	**0.500 (0.033)**	0.372 (0.168)	0.582 (0.144)	0.128 (0.713)	−0.083 (0.855)	−0.211 (0.657)
sMCI Aβ^−^ vs sMCI Aβ^+^	**0.854 (0.014)**	− 0.014 (0.970)	0.210 (0.634)	0.869 (0.092)	0.645 (0.246)	−0.224 (0.699)
pMCI Aβ^−^ vs pMCI Aβ^+^	**1.130 (0.002)**	**0.619 (0.017)**	0.466(0.291)	0.516 (0.371)	0.669 (0.243)	0.153 (0.809)
Associations between neurodegeneration biomarkers and combinations of clinical diagnosis and Aβ pathology						
CN Aβ^−^ vs CN Aβ^+^	**0.500 (0.033)**	0.372 (0.168)	0.582(0.144)	0.128 (0.713)	−0.083 (0.855)	−0.211 (0.657)
CN Aβ^−^ vs sMCI Aβ^+^	**0.716 (0.003)**	0.274 (0.236)	0.055 (0.875)	0.442 (0.181)	0.661 (0.124)	0.219(0.603)
CN Aβ^−^ vs pMCI Aβ^+^	**1.120 (< 0.001**)	**0.941 (< 0.001)**	0.344 (0.191)	0.176 (0.590)	**0.773 (0.032)**	0.597 (0.107)
CN Aβ^−^ vs AD Aβ^+^	**1.100 (< 0.001)**	**0.789 (0.007)**	**0.668 (0.038)**	0.313 (0.378)	0.435 (0.262)	0.121 (0.773)
CN Aβ^−^ vs sMCI Aβ^−^	−0.234 (0.261)	0.244 (0.439)	−0.198 (0.595)	− 0.477 (0.214)	−0.036 (0.934)	0.441 (0.370)
CN Aβ^−^ vs pMCI Aβ^−^	−0.130 (0.560)	− 0.018 (0.959)	−0.264 (0.583)	− 0.112 (0.785)	0.134 (0.800)	0.246 (0.678)

In different combinations of clinical diagnosis and Aβ pathology, data are are estimates from linear mixed-effects models evaluating effects of Aβ within diagnostic groups (top 3 rows), and differences between Aβ^−^ CN and other combinations of diagnosis and Aβ pathology (bottom 6 rows). Results are β-coefficient (*P*-value). Bold values indicate significant associations

*Abbreviations*: *VILIP-1* Visinin-like protein-1, *YKL-40* Chitinase-3-like protein 1, *CN* cognitively normal, *sMCI* stable mild cognitive impairment, *pMCI* progressive mild cognitive impairment, *AD* Alzheimer’s disease

Between CN Aβ- and all other combinations of diagnosis and Aβ pathology, compared to CN Aβ-, p-tau was increased in all Aβ + groups (CN Aβ+, sMCI Aβ+, pMCI Aβ+, and AD Aβ+) (Table 5 and Fig. 3a), and VILIP-1 was increased in pMCI Aβ + and AD Aβ + (Table 5 and Fig. 3b), while YKL-40 was increased in CN Aβ + and AD Aβ + (Table 5 and Fig. 3c).

When comparing the strengths of the associations between CSF biomarkers with different combinations of diagnosis and Aβ pathology, there were no significant differences between p-tau, VILIP-1, and YKL-40. The only exception was that p-tau was more strongly associated with pMCI Aβ + comparing with YKL-40.

### Associations between biomarkers and cognition, brain structure, and WMH

**Fig. 4:**
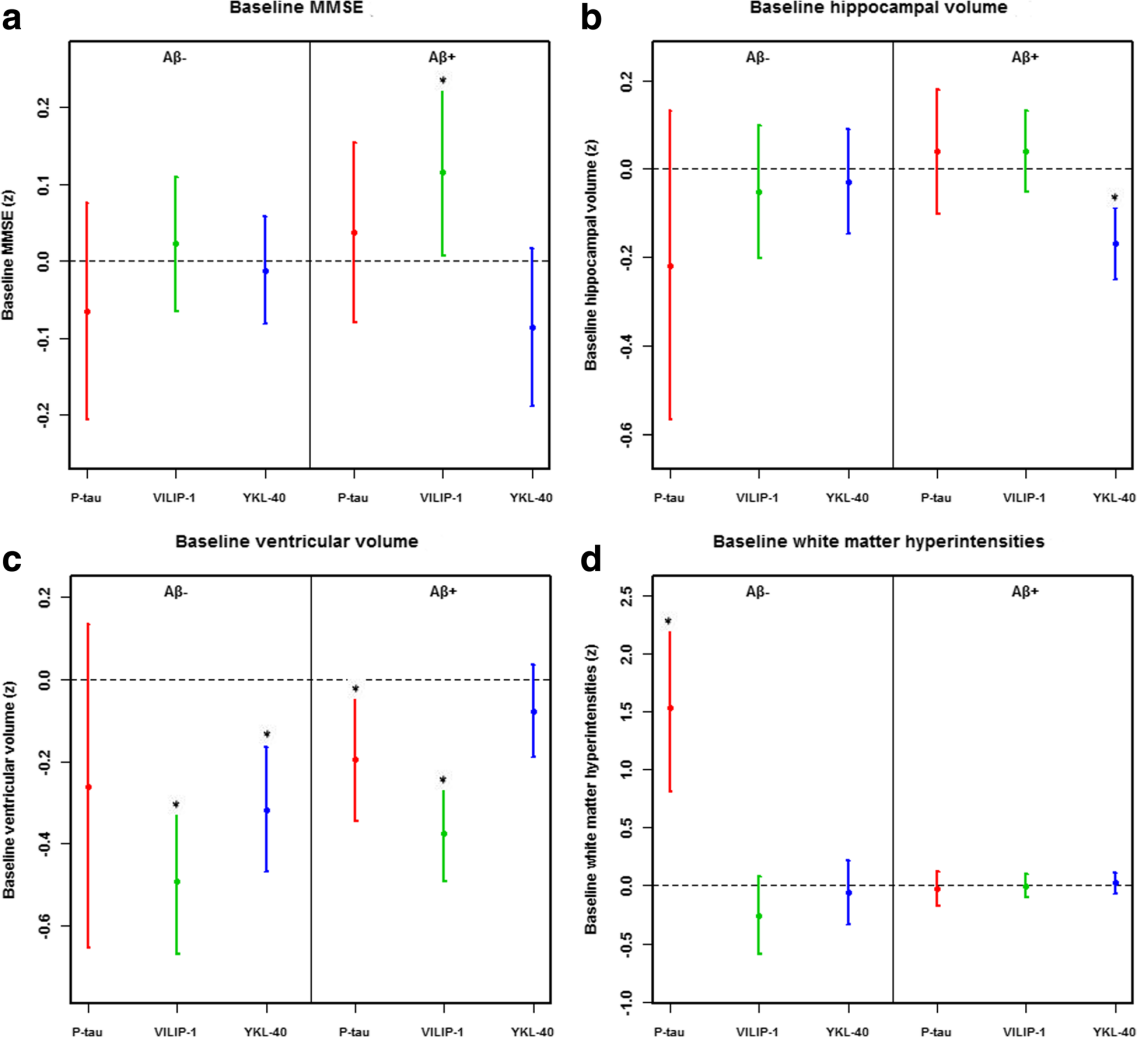
Baseline associations between CSF P-tau, VILIP-1, and YKL-40 with other AD hallmarks. **a**–**d** The data are estimates (β-coefficients) from linear mixed-effects models with a confidence interval of 95%. These estimates are the main effects of biomarkers in the study of the baselines. Effects were significant (*), for MMSE (**a**): VILIP-1 in Aβ^+^ (*p* = 0.037); for hippocampal volume (**b**): YKL-40 in Aβ^+^ (*p* < 0.001); for ventricular volume (**c**): P-tau (*p* = 0.012), VILIP-1 (*p* < 0.001) in Aβ^+^ and VILIP-1 (*p* < 0.001) and YKL-40 (*p* < 0.001) in Aβ^−^ people. For WMH (**d**): P-tau in Aβ^−^ (*p* < 0.001). Biomarkers and outcomes were standardized to z-scores. Abbreviations: VILIP-1, Visinin-like protein-1; YKL-40, Chitinase-3-like protein 1; AD, Alzheimer’s disease

**Fig. 5:**
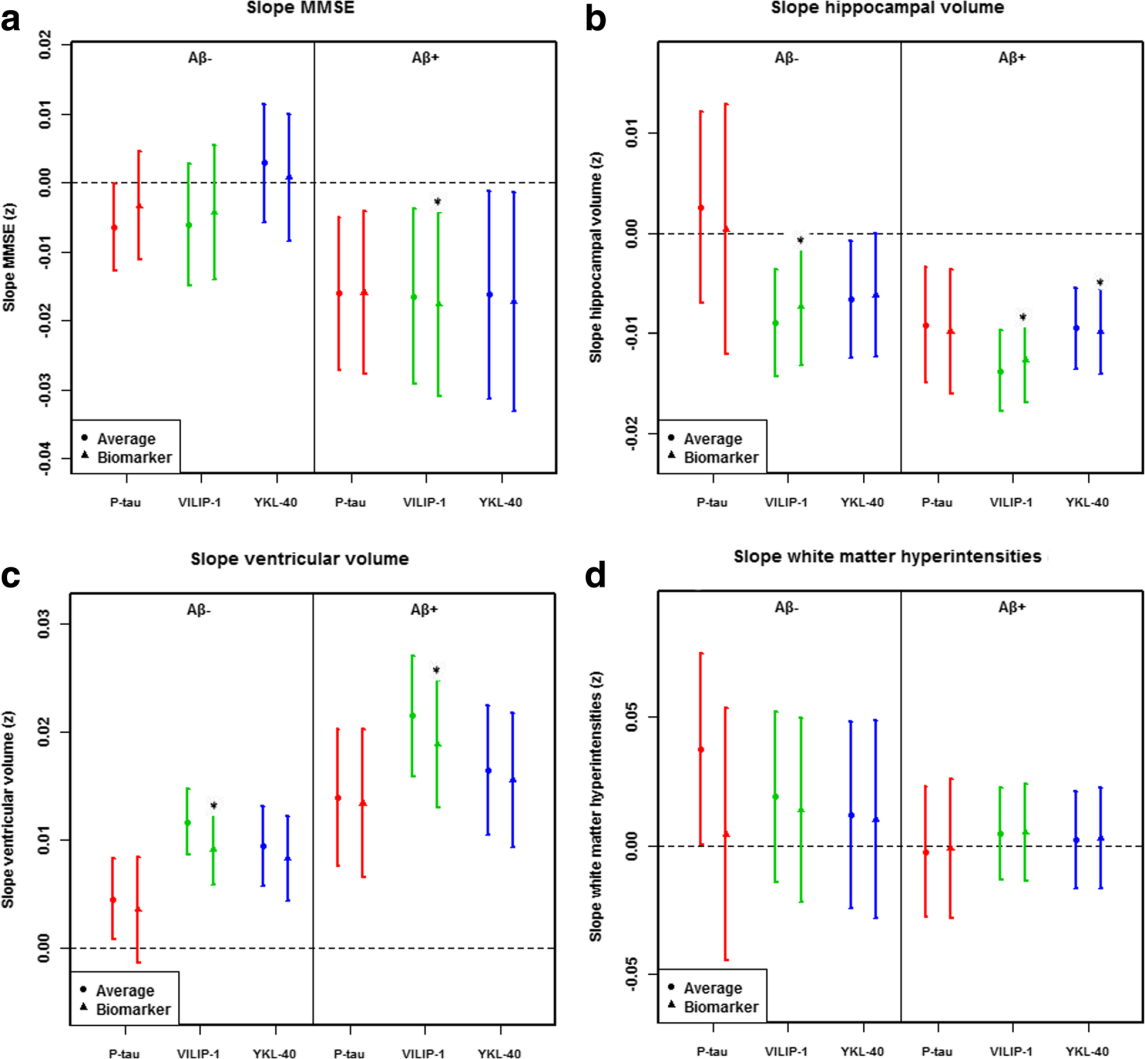
Longitudinal associations between CSF P-tau, VILIP-1, and YKL-40 with other AD hallmarks. **a**–**d** The data are estimates (β-coefficients) from linear mixed-effects models with a confidence interval of 95%. The estimates are the effect of time plus the biomarker by time interactions, capturing the longitudinal effects of the biomarkers. For each model, the “average” effect of time is also displayed for comparison. Effects were significant (*), which means that biomarker levels affected the slopes of the outcome, for MMSE (**a**): VILIP-1 (*p* = 0.033) in Aβ^+^; for hippocampal volume (**b**): VILIP-1 in Aβ^−^ (*p* = 0.041) and Aβ^+^ (*p* < 0.001) and for YKL-40 (*p* = 0.034) in Aβ^+^; for ventricular volume (**c**): VILIP-1 in Aβ^−^ (*p* < 0.001) and Aβ^+^ (*p* < 0.001). Biomarkers and outcomes were standardized to z-scores. Abbreviations: VILIP-1, Visinin-like protein-1; YKL-40, Chitinase-3-like protein 1; AD, Alzheimer’s disease

Baseline and longitudinal data are shown in Fig. 4 and Fig. 5 respectively. At baseline, p-tau was associated with smaller ventricular volumes (in Aβ+) (Fig. 4c) and worsening WMH (in Aβ-) (Fig. 4d), but not with MMSE and hippocampal volumes. Over time, p-tau was not associated with worsening MMSE, hippocampal atrophy, expansion of ventricle volume, and more WMH. At baseline, high VILIP-1 was associated with smaller ventricular volumes (in Aβ- and Aβ+) (Fig. 4c). Over time, VILIP-1 was associated with worsening MMSE (in Aβ+) (Fig. 5a), smaller hippocampal volumes (in Aβ- and Aβ+) (Fig. 5b), and larger ventricles (in Aβ- and Aβ+) (Fig. 5c). At baseline, high YKL-40 was associated with smaller hippocampal (in Aβ+) (Fig. 4) and ventricular (in Aβ-) (Fig. 4c) volumes. Over time, YKL-40 was associated with hippocampal atrophy (in Aβ+) (Fig. 5b).

## Discussion

The present study performed a comprehensive assessment about the characteristic of p-tau, VILIP-1, and YKL-40 of MCI and AD patients from the ADNI database. We have the following main findings: first, the levels of p-tau and VILIP-1 were higher in both pMCI and AD groups compared with CN and sMCI groups. However, the levels of YKL-40 were not significantly different between CN, sMCI, pMCI, and AD participants. Secondly, VILIP-1 and YKL-40 were significant predictors of AD, while p-tau had higher accuracy for single predictors. P-tau and VILIP-1, but not YKL-40, were significant individual predictors for sMCI versus pMCI. Combination of biomarkers did not have higher diagnostic accuracy than the model using p-tau alone for CN versus AD and for sMCI versus pMCI. Our third main finding is that P-tau and VILIP-1 were highly associated with Aβ pathology across clinical stages of AD, while YKL-40 was correlated with Aβ pathology in CN and AD groups. Finally, baseline P-tau was only associated with worsening WMH (in Aβ-). VILIP-1 was associated with acceleration of cognitive decline (in Aβ+), hippocampal atrophy (in Aβ- and Aβ+), and expansion of ventricles (in Aβ- and Aβ+) in longitudinal analyses. YKL-40 was associated with hippocampal atrophy at baseline and follow-up (in Aβ+).

The disorder of calcium homeostasis is considered to be one of the main characteristics of the pathophysiology of AD, and the pathogenesis of AD is closely related to the calcium signaling pathway [27]. Therefore, VILIP-1, as a neuronal calcium sensor protein, might have a connection to the etiology of AD [27]. VILIP-1 is associated with calcium mediated neuronal damage and also participates in the changes in the pathological mechanism of homeostasis, which leads to the loss of neurons [27]. In the present study, the finding of higher levels of VILIP-1 in pMCI and AD compared with CN and sMCI is consistent with previous studies [6–10]. However, another report has found that there is no significant increase of CSF VILIP-1 in patients with AD and MCI [16]. The reasons may be the cognitively normal group (*n* = 37) in that study was biased towards patients who showed decline (6 participants progressed to MCI and 4 participants to dementia) over 4 years, and the rate of APOE ε4 carriership (42%) in cognitively normal group was higher than reported in the previous studies (17–29%) [7, 9]. However, in the current study, we excluded the subjects who were diagnosed as CN at baseline, but converted to MCI or AD during follow-up, and the percentage of APOE ε4 carriership in CN is 15.6%. The increased CSF levels of pMCI and AD patients confirm the utility of VILIP-1 as a useful biomarker of neuronal injury. In this study, we found that the levels of CSF YKL-40 were not significantly increased in MCI and AD compared with CN. This is not consistent with some previous studies [16–21]. The possible reasons are: (1) Levels of YKL-40 in CSF are increased in stroke, multiple sclerosis, amyotrophic lateral sclerosis, and other neurological disorders [28–31], indicating that it is not specific to AD but instead seems to be a more common marker for inflammation; (2) the sample size of this study is small, especially in the AD group.

A previous study indicated that the diagnostic sensitivity of CSF VILIP-1 and VILIP-1/Aβ42 to AD is comparable to CSF tau, p-tau, Aβ42, and tau/Aβ42 or p-tau/Aβ42, respectively [8]. We found that the three biomarkers identified AD versus CN after adjustment for age, gender, and education, while CSF p-tau had higher accuracy than VILIP-1 and YKL-40. Furthermore, combination of biomarkers did not improve the diagnostic accuracy compared to using p-tau alone. Although the triple combination of p-tau, VILIP-1, and YKL-40 had the highest AUC, there were not statistically significant differences compared to p-tau, combination of p-tau and VILIP-1, and combination of p-tau and YKL-40. The combinations of biomarkers also did not increase the proportion of correctly classified people, but it was significantly improved when adjusting the models for demographics and CSF Aβ42, especially only VILIP-1 or YKL-40 and the combination of VILIP-1 and YKL-40. For sMCI versus pMCI, P-tau and VILIP-1 (but not YKL-40) were significant predictors, while the accuracies of sMCI were less than that of pMCI. Similarly, combination wasn’t significantly better than p-tau alone. We speculate biomarkers to be less accurate in sMCI than in pMCI and AD because some sMCI patients may have non-AD disease that reduces biomarker specificity. As shown in Table 3 of this article, unadjusted age, gender, and education, p-tau and VILIP-1 still have diagnostic value for AD and pMCI. After adjusting for age, gender, and education, YKL-40 had diagnostic value for AD, but did not significantly improve the diagnostic value of p-tau and VILIP-1 for AD and pMCI (Additional file 1: Table S1). Therefore, p-tau and VILIP-1 may have clinical value for AD and pMCI individuals.

The onset of AD is a dynamic pathophysiological process, starting from the accumulation of Aβ plaques, continuing with tau pathology, then structural changes in the brain, followed by memory loss, and clinical deterioration [32]. We found that p-tau and VILIP-1 were strongly associated with Aβ pathology and the clinical stage of AD. P-tau was associated with Aβ pathology within all diagnostic groups and had increased levels in CN Aβ+, sMCI Aβ+, pMCI Aβ+, and AD Aβ + compared to CN Aβ-. VILIP-1 was associated with Aβ pathology within pMCI group and had increased levels in pMCI Aβ + and AD Aβ + compared to CN Aβ-. However, YKL-40 was only associated with Aβ-pathology in CN group, and had increased levels in AD Aβ + participants compared to CN Aβ-. All biomarkers were not associated with increased levels in sMCI Aβ- and pMCI Aβ- participants compared to CN Aβ- participants. It is interesting that VILIP-1 was not increased in the CN Aβ + versus CN Aβ-, whereas p-tau and YKL-40 were. Among the correlations between CSF p-tau, VILIP-1, YKL-40, and Aβ42, p-tau has the strongest correlation with VILP-1, therefore, we speculate VILIP-1 may be a disease marker that is downstream to tau pathology.

Our results also demonstrated different relationships between biomarkers and different outcomes. In two longitudinal studies, results suggest that CSF VILIP-1 and VILIP-1/Aβ42 ratio predict future cognitive impairment similarly to tau and tau/Aβ42 ratio [8, 9]. The relationship between YKL-40 and cognitive function is controversial [16, 20]. To the best of our knowledge, there is currently no study of the association of VILIP-1 and YKL-40 with structural MRI and WMH. In this study, VILIP-1 but not YKL-40 was associated with worsening MMSE over time. VILIP-1 primarily had negative associations with ventricle size in Aβ- and Aβ + individuals, which was seen in longitudinal analyses of cognition (in Aβ+), hippocampal atrophy (in Aβ- and Aβ+), and expansion of ventricles (in Aβ- and Aβ+). YKL-40 was associated with hippocampal atrophy (in Aβ+) at baseline and follow-up and negatively associated with ventricle size in Aβ- at baseline. The above results suggest that VILIP-1 and YKL-40 may respond to neurodegeneration in AD. Unexpectedly, p-tau was hardly associated with cognition, hippocampal atrophy, and ventricular expansion at baseline and over time, except for a negative correlation with ventricular size and a correlation with worsening WMH at baseline. The reason for this is unknown, but further studies will be important to clarify the role of p-tau. We found negative correlations between p-tau, VILIP-1, and YKL-40 and ventricle size at baseline. A possible explanation for this is that p-tau, VILIP-1, and YKL-40 not only reflect neuronal damage, but also may be related to the function or transmission of normal neurons, which may lead to the relationship between high biomarker levels and small ventricle volume.

There are limitations to our study. This study did not include non-AD neurodegenerative diseases, and did not have metabolism and neuropathology data. For the sMCI, this study only included participants who were stable for at least 2 years, and this may reduce the number of participants erroneously classified as stable because some participants would have progressed to AD with longer follow-up [33]. In addition, the ADNI database was volunteered by highly educated individuals for research focused on AD research. This may give rise to bias in choice because the study population is a self-selected individual who may have concerns about their cognition. Finally, the self-selectivity of our research population and the relatively small sample size of our research limit the generality of our findings to the wider community. Therefore, our findings will need to be validated in a larger population-based cohort.

## Conclusions

In summary, our results show that VILIP-1 or YKL-40 is not better than P-tau in the diagnostic accuracy for AD and MCI, and combinations of p-tau, VILIP-1, and YKL-40 do not increase the diagnostic accuracy for CN versus AD and for sMCI versus pMCI, while p-tau and VILIP-1 have correlations to Aβ pathology and the clinical stages of AD, and VILIP-1 and YKL-40 may respond to neurodegeneration in AD.

## Additional file

Additional file 1: **Table S1.** Diagnostic accuracy of CSF P-tau, VILIP-1, and YKL-40 (Adjusted for age, gender, education). For AUC, the letters a-g indicate significant differences versus other models: P-tau (a), VILIP-1 (b), YKL-40 (c), P-tau & VILIP-1 (d), P-tau & YKL-40 (e), VILIP-1 & YKL-40 (f), P-tau & VILIP-1 & YKL-40 (g). Bold values indicate significant associations. Abbreviations: VILIP-1, Visinin-like protein-1; YKL-40, Chitinase-3-like protein 1; CN, cognitively normal; sMCI, stable mild cognitive impairment; pMCI, progressive mild cognitive impairment; AD, Alzheimer’s disease. (DOC 18 kb)

## Abbreviations

AD: Alzheimer’s disease
ADNI: Alzheimer’s disease Neuroimaging Initiative
ANOVA: Analysis of covariance
AUC: Area under the receiver operator characteristics curve
Aβ: Amyloid-β
CN: Cognitively normal
CSF: Cerebrospinal fluid
MCI: Mild cognitive impairment
MMSE: Mini-mental State Examination
MRI: Magnetic resonance imaging
PET: Positron emission tomography
ROC: Receiver operating curve
VILIP-1: visinin-like protein-1
WMH: white matter hyperintensity
YKL-40: chitinase-3-like protein 1

## References

[1] Blennow K, de Leon MJ, Zetterberg H (2006). Alzheimer’s disease. Lancet.

[2] Perl DP (2010). Neuropathology of Alzheimer’s disease. Mt Sinai J Med.

[3] Jack CR, Bennett DA, Blennow K, Carrillo MC, Feldman HH, Frisoni GB (2016). A/T/N: an unbiased descriptive classification scheme for Alzheimer disease biomarkers. Neurology.

[4] Gozes I (2017). Specific protein biomarker patterns for Alzheimer’s disease: improved diagnostics in progress. EPMA J.

[5] Lista S, Zetterberg H, Dubois B, Blennow K, Hampel H (2014). Cerebrospinal fluid analysis in Alzheimer’s disease: technical issues and future developments. J Neurol.

[6] Mroczko B, Groblewska M, Zboch M, Muszynski P, Zajkowska A (2015). Evaluation of visinin-like protein 1 concentrations in the cerebrospinal fluid of patients with mild cognitive impairment as a dynamic biomarker of Alzheimer’s disease. J Alzheimers Dis.

[7] Luo X, Hou L, Shi H, Zhong X, Zhang Y, Zheng D (2013). CSF levels of the neuronal injury biomarker visinin-like protein-1 in Alzheimer’s disease and dementia with Lewy bodies. J Neurochem.

[8] Tarawneh R, D'Angelo G, Macy E, Xiong C, Carter D, Cairns NJ (2011). Visinin-like protein-1: diagnostic and prognostic biomarker in Alzheimer disease. Ann Neurol.

[9] Tarawneh R, Lee JM, Ladenson JH, Morris JC, Holtzman DM (2012). CSF VILIP-1 predicts rates of cognitive decline in early Alzheimer disease. Neurology.

[10] Babic Leko M, Borovecki F, Dejanovic N, Hof PR, Simic G (2016). Predictive value of cerebrospinal fluid visinin-like protein-1 levels for Alzheimer’s disease early detection and differential diagnosis in patients with mild cognitive impairment. J Alzheimers Dis.

[11] Heppner FL, Ransohoff RM, Becher B (2015). Immune attack: the role of inflammation in Alzheimer disease. Nat Rev Neurosci.

[12] Achilli C, Ciana A, Minetti G (2018). Brain, immune system and selenium: a starting point for a new diagnostic marker for Alzheimer's disease?. Perspect Public Health.

[13] Allen HB (2016). Alzheimer’s disease: assessing the role of spirochetes, biofilms, the immune system, and amyloid-beta with regard to potential treatment and prevention. J Alzheimers Dis.

[14] Perrin RJ, Craig-Schapiro R, Malone JP, Shah AR, Gilmore P, Davis AE (2011). Identification and validation of novel cerebrospinal fluid biomarkers for staging early Alzheimer's disease. PLoS One.

[15] Fagan AM, Perrin RJ (2012). Upcoming candidate cerebrospinal fluid biomarkers of Alzheimer’s disease. Biomark Med.

[16] Kester MI, Teunissen CE, Sutphen C, Herries EM, Ladenson JH, Xiong C (2015). Cerebrospinal fluid VILIP-1 and YKL-40, candidate biomarkers to diagnose, predict and monitor Alzheimer’s disease in a memory clinic cohort. Alzheimers Res Ther.

[17] Craig-Schapiro R, Perrin RJ, Roe CM, Xiong C, Carter D, Cairns NJ (2010). YKL-40: a novel prognostic fluid biomarker for preclinical Alzheimer’s disease. Biol Psychiatry.

[18] Olsson B, Hertze J, Lautner R, Zetterberg H, Nagga K, Hoglund K (2013). Microglial markers are elevated in the prodromal phase of Alzheimer’s disease and vascular dementia. J Alzheimers Dis.

[19] Rosen C, Andersson CH, Andreasson U, Molinuevo JL, Bjerke M, Rami L (2014). Increased levels of chitotriosidase and YKL-40 in cerebrospinal fluid from patients with Alzheimer’s disease. Dement Geriatr Cogn Dis Extra.

[20] Hellwig K, Kvartsberg H, Portelius E, Andreasson U, Oberstein TJ, Lewczuk P (2015). Neurogranin and YKL-40: independent markers of synaptic degeneration and neuroinflammation in Alzheimer’s disease. Alzheimers Res Ther.

[21] Janelidze S, Hertze J, Zetterberg H, Landqvist Waldo M, Santillo A, Blennow K (2016). Cerebrospinal fluid neurogranin and YKL-40 as biomarkers of Alzheimer’s disease. Ann Clin Transl Neurol.

[22] Mattsson N, Tabatabaei S, Johansson P, Hansson O, Andreasson U, Mansson JE (2011). Cerebrospinal fluid microglial markers in Alzheimer’s disease: elevated chitotriosidase activity but lack of diagnostic utility. NeuroMolecular Med.

[23] Tierney MC, Fisher RH, Lewis AJ, Zorzitto ML, Snow WG, Reid DW (1988). The NINCDS-ADRDA work group criteria for the clinical diagnosis of probable Alzheimer’s disease: a clinicopathologic study of 57 cases. Neurology.

[24] Shaw LM, Vanderstichele H, Knapik-Czajka M, Clark CM, Aisen PS, Petersen RC (2009). Cerebrospinal fluid biomarker signature in Alzheimer’s disease neuroimaging initiative subjects. Ann Neurol.

[25] Risacher SL, Saykin AJ (2013). Neuroimaging and other biomarkers for Alzheimer’s disease: the changing landscape of early detection. Annu Rev Clin Psychol.

[26] Schwarz C, Fletcher E, DeCarli C, Carmichael O (2009). Fully-automated white matter hyperintensity detection with anatomical prior knowledge and without FLAIR. Inf Process Med Imaging.

[27] Groblewska M, Muszynski P, Wojtulewska-Supron A, Kulczynska-Przybik A, Mroczko B (2015). The role of visinin-like protein-1 in the pathophysiology of Alzheimer’s disease. J Alzheimers Dis.

[28] Hjalmarsson C, Bjerke M, Andersson B, Blennow K, Zetterberg H, Aberg ND (2014). Neuronal and glia-related biomarkers in cerebrospinal fluid of patients with acute ischemic stroke. J Cent Nerv Syst Dis.

[29] Magdalinou NK, Paterson RW, Schott JM, Fox NC, Mummery C, Blennow K (2015). A panel of nine cerebrospinal fluid biomarkers may identify patients with atypical parkinsonian syndromes. J Neurol Neurosurg Psychiatry.

[30] Martinez MA, Olsson B, Bau L, Matas E, Cobo Calvo A, Andreasson U (2015). Glial and neuronal markers in cerebrospinal fluid predict progression in multiple sclerosis. Mult Scler.

[31] Winer L, Srinivasan D, Chun S, Lacomis D, Jaffa M, Fagan A (2013). SOD1 in cerebral spinal fluid as a pharmacodynamic marker for antisense oligonucleotide therapy. JAMA Neurol.

[32] Jack CR, Knopman DS, Jagust WJ, Shaw LM, Aisen PS, Weiner MW (2010). Hypothetical model of dynamic biomarkers of the Alzheimer’s pathological cascade. Lancet Neurol.

[33] Mattsson N, Insel PS, Palmqvist S, Portelius E, Zetterberg H, Weiner M (2016). Cerebrospinal fluid tau, neurogranin, and neurofilament light in Alzheimer’s disease. EMBO Mol Med.

