# Supplementary material for: Cerebrospinal fluid phosphorylated tau, visinin-like protein-1, and chitinase-3-like protein 1 in mild cognitive impairment and Alzheimer’s disease
Source: Transl Neurodegener. 2018 Sep 10;7:23. doi: 10.1186/s40035-018-0127-7 (PMC6161434; doi:10.1186/s40035-018-0127-7)
Supplement: Supplementary file 1 — Table S1. Diagnostic accuracy of CSF P-tau, VILIP-1, and YKL-40 (Adjusted for age, gender, education). For AUC, the letters a-g indicate significant differences versus other models: P-tau (a), VILIP-1 (b), YKL-40 (c), P-tau & VILIP-1 (d), P-tau & YKL-40 (e), VILIP-1 & YKL-40 (f), P-tau & VILIP-1 & YKL-40 (g). Bold values indicate significant associations. Abbreviations: VILIP-1, Visinin-like protein-1; YKL-40, Chitinase-3-like protein 1; CN, cognitively normal; sMCI, stable mild cognitive impairment; pMCI, progressive mild cognitive impairment; AD, Alzheimer’s disease. (DOC 18 kb) [file 40035_2018_127_MOESM1_ESM.doc]

Table S1 Diagnostic accuracy of CSF P-tau, VILIP-1, and YKL-40 (Adjusted for age, gender, education).

| Groups |  | Model P-tau VILIP-1 YKL-40 AUC (95% CI) |
| --- | --- | --- |
| CN  vs  AD |  | P-tau only **1.234** 0.944 (0.885-1.004) ^b,c,f^  **(p= 0.001)** |
|  |  | VILIP only  **1.028** 0.814 (0.696–0.933) ^a,c,d,e,g^  **(p=0.003)** |
|  |  | YKL-40 only **1.005** 0.700 (0.543–0.856) ^a,b,d,,e,f,g^  **(p=0.048)** |
|  |  | P-tau & VILIP **1.222** 1.010 0.937 (0.872–1.003) ^b,c,f^  **(p=0.002)** (p=0.521) |
|  |  | P-tau & YKL-40 **1.231** 1.002 0.936 (0.870–1.001) ^b,c,f^  **(p= 0.001)** (p=0.666) |
|  |  | VILIP & YKL-40 **1.026** 0.000 0.806 (0.681–0.930) ^a,c,d,e,g^  **(p=0.008)** (p=0.501) |
|  |  | P-tau & VILIP & YKL-40 **1.221** 1.009 1.001 0.941 (0.878–1.004) ^b,c,f^  **(p= 0.002)** (p=0.559) (p=0.735) |
| sMCI  vs  pMCI |  | P-tau only **1.053**  0.771 (0.656–0.886) ^c^  **(p= 0.013)** |
|  |  | VILIP only **1.011** 0.746 (0.629–0.864)  **(p= 0.033)** |
|  |  | YKL-40 only 1.002 0.686 (0.560–0.813) ^a,d,,e,f,g^  (p= 0.451) |
|  |  | P-tau & VILIP 1.041 1.006 0.777 (0.667–0.888) ^c^  (p= 0.086) (p=0.314) |
|  |  | P-tau & YKL-40 **1.056** 0.999 0.766 (0.652–0.880) ^c^  **(p= 0.017)**  (p= 0.751) |
|  |  | VILIP & YKL-40 **1.012** 1.000 0.745 (0.627–0.862)  **(p=0.044)** (p=0.946) |
|  |  | P-tau & VILIP & YKL-40 1.044 1.007 0.999 0.772 (0.661–0.883) ^c^  (p=0.076) (p=0.275) (p=0.586) |

For AUC, the letters a-g indicate significant differences versus other models: P-tau (a), VILIP-1 (b), YKL-40 (c), P-tau & VILIP-1 (d), P-tau & YKL-40 (e), VILIP-1 & YKL-40 (f), P-tau & VILIP-1 & YKL-40 (g). Bold values indicate significant associations. Abbreviations: VILIP-1, Visinin-like protein-1; YKL-40, Chitinase-3-like protein 1; CN, cognitively normal; sMCI, stable mild cognitive impairment; pMCI, progressive mild cognitive impairment; AD, Alzheimer’s disease.
